# Measurement of Quality of Life VII. Statistical Covariation and Global Quality of Life Data: The Method of Weight-Modified Linear Regression

**DOI:** 10.1100/tsw.2003.89

**Published:** 2003-10-13

**Authors:** Soren Ventegodt, Joav Merrick

**Affiliations:** The Quality of Life Research Center, Copenhagen K, Denmark

**Keywords:** quality of life, QOL, SEQOL, QOL5, QOL1, measurement, statistics, human development, holistic medicine, public health, Denmark

## Abstract

Existing standard statistical procedures do not seem to fulfill the needs of the researcher in global quality-of-life (QOL) research, because the most interesting question seems to be the exact size of statistical covariations. A method is necessary if we are to isolate the most important factors connected to quality of life among the thousands of possible factors in life. We have developed a new procedure we call “weight-modified linear regression”. Unfortunately as demonstrated in the discussion, the procedure is not totally without problems and weaknesses. In spite of the critique, we believe the procedure to be valid for the purpose of estimating the size of the covariation in population studies including psychometric measures of global quality of life. As we need to be certain that the procedure is valid, we hereby invite the scientific community to give us further critique of the method and suggestions for its improvement.

## INTRODUCTION

Over the years, countless statistical procedures have been developed, most of them extremely successful, but when it comes to psychometrics there are still problems. Just as we still face severe problems with the scales when trying to collect our psychometric data[1,2], it is still not that simple to determine the absolute extent of the statistical covariation from such data. We have not found a traditional method that elegantly solves the problem of how large the statistical covariation is between two variables in absolute terms, when one or both of these variables is a global quality-of-life (QOL) measure.

The difficulty arises when a plot including a psychometric measure of global quality of life gives a swarm of dots (x,y) with a very irregular distribution. This is often the case, as the answers "extremely bad" and "bad" from the bottom of a 5-point quality-of-life Likert scale may be rare. Often an extremely low quality-of-life rating will be related to a short-lived condition characterized by huge efforts in improving quality of life. There is often an excessive mortality rate for the group of people feeling "extremely bad". An experienced condition deemed worse than death is not stable, and failure to solve problems within a short time-span will often lead to suicide. This dynamic makes the group with the lowest quality of life very small, sometimes only 1% of the population.

Low global quality of life is statistically connected to poor health[3]. In accordance with this, the life mission theory predicts a very high prevalence of sickness for the group with a very low global quality of life[4]. To understand the nature of life crises, we have to understand the structure of the ego. In order to radically improve global quality of life, it seems necessary to have a fundamental transformation of the psyche. Such a shift in personality has been labeled an "ego death" in Buddhism[5] or a psychic death by Jung[6,7], because it implies a shift back to the existential position of the natural self, i.e., living the true purpose of life[4]. The problem of healing and improving the global quality of life seems strongly connected to the unpleasantness of the ego-death experience, and often a person who lacks the understanding necessary for personal development chooses death instead of personal transformation. The quality-of-life peak experience, which is typically described as moments of total being, great clarity, intense happiness, and deep understanding of life, occurs spontaneously for only a few percent of the population[8].

It is therefore not difficult to understand why population surveys including psychometric variables of global quality of life have an extreme low representation of individuals with very low quality of life. As the commonly used statistical methods do not put a special focus on persons in the rare groups, these groups often drown in very huge and statistically dominating center groups, sometimes making the connections seem smaller than they are.

As an example, we can use the statistical covariation between global quality of life on one side and the positive/negative way of viewing life on the other side. In the Copenhagen Perinatal Birth Cohort 1959–61, 4,589 persons answered both the questions on quality of life and the questions on the view of life (see Table 1 at end of paper)[9].

Only 10 of the respondents did actually have an extreme negative view of life. A linear regression would have been completely blind towards 0.2% of the respondents in this group. The variation in the interval of measurement — the difference between the best and the worst group — was 58.8%, with the eighth and most negative group responsible for almost one-sixth of this difference. In the dimension of immediate subjective well being, this eighth extreme group was responsible for one-quarter of the total difference in the interval.

Now, to establish the uncertainty of the measuring and the real size of the interval, we needed a suitable statistical tool to help us determine the extent of this statistical covariation, where we have global quality of life on the one axis, and a similar psychometric variable, view of life, on the other. To resolve this problem we have developed a method we call "weight-modified linear regression".

If we use the formula for weight-modified linear regression, which follows below after a short discussion of the strengths and weaknesses of the most commonly used statistical procedures, the extent of covariation was determined to 60.0 +/- 4.4% (significance level: $p = 0.05$). The extent of the covariation between the view of life measured by 21 questions in the SEQOL, and the global (total) quality of life, is at least 55.6%.

## THE TRADITIONAL METHODS

Pearson's correlation coefficient, normally labeled r, is one of the most widely used statistical tools for measuring association. The presumption is linearity, and the method also gives the statistical significance ($p$) associated with rejecting the null hypothesis that the population correlation is zero. We do not hereby have an expression for the extent of the covariance in absolute terms (percentages) and therefore we are not able to judge on the clinic relevance in patient-studies or the significance of a factor in a population survey.

Linear regression describes the most plausible straight line through the swarm of dots (x,y), and correspondingly indicates the significance. From this line we can calculate the variation over the measured interval. However, in a very distorted distribution as described above, the small percentage of dots representing people who have given answers in the extremely low categories may be far from the regression line dominated by the majority, and valuable information may be lost.

We discovered our problem when we could not find the same results by these standard methods, as we found by simply measuring a drawn curve. Most often the calculated covariation was too small because of a nonlinear tendency in the extreme end of the interval.

We then searched for an explanation of the problem and found that it was necessary to take the structure of the answering scale into consideration. To find a better expression of the covariation (x vs. y) we first calculated the average of the answers y for each possible category of the Likert scale (for each of the Likert scale point groups, please see the formalism below), and then ran the regression using these new points, giving them *equal weight* (hence the name of the procedure). There is a problem if the number in the group comes close to zero. Another problem is that the extreme answers sometimes arise from people making fun of the survey or from mentally ill persons not reporting their quality-of-life state fairly. Using the formula in a practical way, we have chosen the minimum number in an answer-group to be four answers.

Below follows the mathematical formalism of this procedure that we have named "weight-modified linear regression". The original idea to the solution was originally formulated by Hilden (the mathematical formalism) and Ventegodt[10].

## WEIGHT-MODIFIED LINEAR REGRESSION (TREND-ANALYSIS, MODIFIED REGRESSION)

The purpose of the special weighted regression used in the Copenhagen Quality of Life study was to measure the linear component, possibly in a curved line relationship between a factor *x* and a quality of life measure *y,* and to do it in such a way that the special relationships in the interesting minority groups, that is, those with a particularly high or low *x* value, are revealed at the cost of possible flat (horizontal) or opposing trends in the central part of the *x* distribution. This assumes that the minority groups are not too small, in which case they are amalgamated with the neighboring group.

This approach is only useful when x can only take on a few values, $x_1$, $x_2$,... $x_k$ (typically, as in QOL1[11]) $k = 5$ and $x_1 = 0.1$, $x_2 = 0.3$, $x_3 = 0.5$, $x_4 = 0.7$, and $x_5 = 0.9$ for our use with the five-point Likert scale; if the result is calculated from a calculated mean of say five Likert scales (as in QOL5[11]), the number *k* will be 25. Let $n_1$, $n_2$,...,$n_k$ be the number of observations in each of the k *x*-groups (for example: $n_2 = 84$ signifies that 84 people answered $x_2 = 0.3$ = "poor"). The slope is defined as

$$h = \frac{\sum_j \mu_j (x_j - x_m)}{\sum_j (x_j - x_m)^2}, \quad (1)$$

where $\mu_j$ (for consistency with the notation for x one could prefer $y_{mj,}$ or alternatively, the Greek letter nu [ν]) is the median value of *y* in the *j*th *x*-group, and $x_m$ is the median *x*-value, established by the condition

$$\sum_j (x_j - x_m) = 0. \quad (2)$$

In order to understand the formula for $h$ it might be useful to note that if $\mu_1 = a$ (a constant), then $h = 0$; and if $\mu_j = bx_j$ (that is, it is proportional to $x_j$), then $h = b$ (the proportionality constant). If $\mu_j$ varies linearly with $x_j$, then $\mu_j = a + bx_j$, since $h$ is again equal to $b$, which expresses the average change in the quality of life measure $y$, when $x$ increases by one unit. If $\mu_j$ varies nonlinearly, as a function of $x_j$, $h$ can also be regarded as the slope of a linearized variation of y with $x$.

If $\bar{y}_j$ describes the estimated $\mu_j$, which is equal to the average of the $n_j$ $y$-values of people who answered $x_j$, then $h$ is estimated as

$$est\ h = \frac{\sum_j \bar{y}_j (x_j - x_m)}{S}, \tag{3}$$

where S is the same denominator as in (Eq. 1). On multiplying by ($x_k$-$x_2$), we obtain the estimated increase in y from the lowest to the highest ($k$'th) $x$-group (using the linearized form if $y$ is a nonlinear function of $x$). This is denoted as Δ2:

$$\Delta 2 = (x_k - x_1)\,(est\ h). \tag{4}$$

The difference between the highest and lowest groups of $\bar{y}\,1_j$ are denoted by

$$\Delta 1 = (\max_{j=1,\ldots k} \bar{y}_j) - (\min_{j=1,\ldots k} \bar{y}_j). \tag{5}$$

The quantity Δ1 will tally with Δ2, *if* the $\bar{y}\,2_j$ depend more or less linearly on $x_j$; otherwise Δ1 will reveal a significant departure from linearity. The uncertainty in Δ1 is large and difficult to estimate. On the other hand, the uncertainty in Δ2 is easy to calculate (the following section assumes familiarity with the basic calculation of the standard error).

The standard error (SE) associated with Δ2 is, regardless of the degree of linearity and normality, equal to the square root of

$$SE^2\{\Delta 2\} = (x_k - x_1)^2 \frac{\sum_j SE^2\{\bar{y}_j\}(x_j - x_m)^2}{S^2}, \tag{6}$$

where

$$SE^2\{\bar{y}_j\} = \frac{(SD_j)^2}{n_j}. \tag{7}$$

Here $SD_j$ is the standard deviation in the $y$-distribution of the $x_j$. For our purposes, we can as a rule assume that the variability of $y$ is the same in all the $k$ $x$-groups, that is, $SD_1$ = SD2 =......= $SD_k$. The common SD is then estimated naturally as the square root of

$$s^2 = \frac{\sum_j SAK_j}{\sum_j (n_j - 1)}, \qquad (8)$$

where $SAK_j$ is the relevant sum of squared deviations,

$$SAK_j = \sum_{i=1}^{n_j} (y_{ij} - \bar{y}_j)^2, \qquad (9)$$

where $y_{ij}$ refers to the i'th person's $y$-value of the $n_j$ people who responded to $x_j$.

The values signified as ±ε in connection with Δ2 in the tables are

$$\pm\varepsilon = \pm 1.96 \times SE\{\Delta 2\} \qquad (10)$$

The quantity ε is interpreted as follows: there is a 95% certainty that Δ2 is estimated with an error of less than plus or minus ε.

## DISCUSSION

Jørgen Hilden has lately given important comments on this method[12]. They can be divided into three points:

- "Even though the procedure seemed reasonable for the purpose that this project have made of it, we need a much more profound argumentation in order to convince the readers, or in other words that it extracts from the data exactly what is interesting, with respect to the quality of life. Put in another way it ignores the little movements in the huge center-groups and accentuates the tendency in the small outer-groups to exactly the extent that we are interested in."

An appropriate answer to this just critique seems to be, that seen philosophically, all the groups corresponding to the Likert scales points are equally interesting. Therefore it seems quite reasonable to give them the same importance in the interpretation, irrespective of the absolute number of participants in each group, which is exactly what the proposed procedure does.

- "A related question is the following: It is obvious that you cannot use the procedure to anything intelligent if the outer-groups are very small (this caution is also discussed in the text above). But the question is: how much is "very" small? N = 2,5,10,25 or how much? And does the answer rely on the total N?

An operational rule must be added to the procedure, and this has to be based on objective criteria. The problem here is also, that the procedure is not derived from a conceptual criteria. Proper statistical procedures are derived from a desiderata of the form: "we wish to quantify in a certain degree..." (i.e., "we wish to quantify the next patient's increase in chance of being cured by shifting from placebo to medicament XYZ"), and there one must derive through mathematical analysis the effect-estimate that, with as little bias as possible and with as little standard error as possible, will increase the amount desired. It is not enough with a vague desire like "we wish to measure the rise in the quality of life in a way that stresses the outer-groups". As it is, the procedures remain ad hoc and theoretically uninteresting."

- “There is also a problem with the terminology, since statistical rules ignore any of the Likert scale point groups as they all carry the same meaning and importance.”

Hilden[12] gives an important argument against the procedure. But we believe that we actually are using a conceptual criterion: when we philosophically state that all the Likert scale point groups are of equal interest, this might be wiser that it seems. To go to one extreme: what if there is only one point in a group? It seems intuitively meaningless to ascribe to one point in the group “very low quality of life” the same statistic meaning as to 1,000 points or answers in a center-group. But if we truly want to understand the profound concept of human quality of life, the single person from group five really is as interesting as the 1,000 persons from group two. He or she simply is our only source to knowledge of that state of being. Compare the well-known example of one cancer patient in a thousand having a complete, spontaneous remission. This patient might hold the key to healing, so this patient can be considered as of similar interest as the other 999 not recovering.

The conclusion is, that because of the special conditions like a possible nonlinearity in the extreme ends of the quality-of-life scales, it seems philosophically much more meaningful to extract all the possible knowledge from just one or a few answers in an extreme quality-of-life group, than to try to extrapolate to these extreme groups from the variation among the central groups. Only a human being who has lived the extreme can report on extreme states of being.

From time to time, respondents will appear who are not very serious or who consciously choose to obstruct the survey by giving impossible or untrue answers. Most of such nonvalid questionnaires are easily spotted by their weird characteristics (i.e., the answer “5” to 100 questions in a row) and can be removed in the routine qualitative inspection of the incoming questionnaires in a survey. This problem seems to be fairly eliminated if we take at least four respondents.

Another question, that we have raised ourselves is if we have to extend this linear method into the nonlinear area, as the quality-of-life curves often lose their linearity in the extreme ends of the global quality-of-life scales. Interestingly, this problem seems also to be minimized with weight-modified linear regression, as the low-quality-of-life Likert scale point group’s “jumps of the line” seems to be well represented in the results. Further research is needed to estimate the size of this problem.

Questions might also be raised if grouped values are used for the analysis. Malcolm Campbell[13] suggests that “in this situation, one may have to treat the two (grouped) variables as ordinal and estimate association using Kendall's tau correlation or Spearman's rho correlation. Another possibility is that the chi-square test for trend is used: this is applied to tables where either both row and column variables are ordinal or one is ordinal and the other dichotomous (having two categories), and it is closely related numerically to Pearson's correlation. Bland[14] discusses the test and gives a formula for the test statistic without explicitly stating that his test statistic is the number of cases multiplied by the Pearson correlation between the two variables. The formula used by SPSS is (N-1) * r - they call their test the Mantel-Haenzsel test for linear association (i.e., trend) — see http://www.spss.com/tech/stat/Algorithms/11.5/crosstabs.pdf. Bland[14] points out that the values on the row and columns are usually taken to be 1, 2, 3..., but that other values could be used to reflect the relative importance of the different categories. Kendall's and Spearman's correlations might also "ignore" the interesting outlying cases, but choosing appropriate values for the rows and columns may give them more weight. Choices for the values may have to be ad hoc, and it would be necessary to experiment with different sets of values to observe the effect, but even then, the approach only comes up with a test and not an effect size. Correlation coefficients are effect sizes, but as mentioned above, the nonparametric correlations may well "miss" the cases with small scores. Perhaps that is also true of reporting means and medians: the emphasis is on measuring and reporting the average at the expense of the very small or very large.” We are grateful for these remarks from Campbell.

## CONCLUSIONS

The existing statistical standard procedures do not seem to fulfill the needs of the research in global quality of life, because the most interesting question seems to be the exact size of statistical covariations. This is necessary if we are to isolate the most important factors connected to quality of life, among the thousands of possible factors in life. We have developed a new procedure we call "weight-modified linear regression". Unfortunately as demonstrated in the discussion, the procedure is not totally without problems and weaknesses.

When weight-modified linear regression is used in practice, the researcher would always have to perform and report the usual Pearson correlation (or linear regression) analyses for comparison and in order to see the difference between the two. He or she should report findings in the interesting (small-score) subgroups in any case.

In spite of the critique, we believe the procedure to be valid for the purpose of estimating the size of the covariation in population studies including psychometric measures of global quality of life. As we need to be certain that the procedure is valid, we hereby invite the scientific community to give us further critique of the method and suggestions for its improvement.

## ACKNOWLEDGMENTS

This report presented results from the Danish Quality of Life Population Survey, the planning and execution of which would not have been possible without the aid of Dorte Loldrup Poulsen, Jørgen Hilden, and the late Bengt Zachau-Christiansen. Funds were received from nine Danish Foundations, including The 1991 Pharmacy, the Goodwill, the JL, E. Danielsens & Wife's, Emmerick Meyer's, the Frimodt-Heineken, the Hede Nielsen Family, Petrus Andersen's, C.P. Frederiksen's and the Wedell-Wedellsborgs Foundations, and IMK Almene Fond. The research was approved by the Copenhagen Ethical Committee under numbers (KF) V 100.1762/90 and (KF) 01-502/93.

## BIOSKETCHES

**Søren Ventegodt, MD,** is the Director of the Quality of Life Research Center in Copenhagen, Denmark. He is also responsible for a Research Clinic for Holistic Medicine in Copenhagen and is a popular speaker throughout Scandinavia. He has published numerous scientific or popular articles and a number of books on holistic medicine, quality of life, and quality of working life. His most important scientific contributions are the comprehensive SEQOL questionnaire, the very short QOL5 questionnaire, the integrated quality-of-life (IQOL) theory, the holistic process theory, the life mission theory, and the Danish Quality of Life Research Survey, 1991–94 in cooperation with the University Hospital of Copenhagen and the late pediatric professor Bengt Zachau-Christiansen. E-mail: ventegodt@livskvalitet.org. Website: www.livskvalitet.org/

**Joav Merrick, MD, DMSc,** is Professor of Child Health and Human Development affiliated with the Zusman Child Development Center and Division of Community Health at the Ben Gurion University, Beer-Sheva, Israel and presently the Medical Director of the Division for Mental Retardation, Ministry of Social Affairs, Jerusalem and the Director of the National Institute of Child Health and Human Development. He has numerous publications in the field of child and human development, rehabilitation, intellectual disability, disability, health, welfare, abuse, advocacy and prevention. Dr. Merrick received the Peter Sabroe Child Award for outstanding work on behalf of Danish Children in 1985 and the International LEGO-Prize (“The Children’s Nobel Prize”) for an extraordinary contribution towards improvement in child welfare and well being in 1987. E-mail: jmerrick@internet-zahav.net. Website: www.nichd-israel.com

## TABLE 1
## When the View of Life (Positive vs. Negative) is Plotted vs. the Global Quality of Life, the Nonlinear Nature of the Covariation becomes Apparent (see text) (Note the Distorted Distribution Away from the Low Quality of Life)

### Table of Frequencies

| | | | | | 1 | | | | | 2 | | | | | 3 | | | | | 4 | | | | |
|---|---|---|---|---|---|---|---|---|---|---|---|---|---|---|---|---|---|---|---|---|---|---|---|---|
| | | | **No. of Resp.** | **Score** | **10** | **30** | **50** | **70** | **90** | **10** | **30** | **50** | **70** | **90** | **10** | **30** | **50** | **70** | **90** | **10** | **30** | **50** | **70** | **90** |
| 1 | Positive | 18.5–21 | 1358 | 8 | 0 | 3 | 22 | 497 | 836 | 0 | 2 | 24 | 580 | 751 | 0 | 1 | 113 | 706 | 537 | 0 | 1 | 23 | 710 | 568 |
| 2 | ……….. | 16.5–18 | 1473 | 7 | 1 | 9 | 90 | 827 | 545 | 0 | 22 | 124 | 913 | 412 | 0 | 7 | 363 | 811 | 289 | 1 | 4 | 112 | 930 | 312 |
| 3 | ……….. | 13.5–16 | 813 | 6 | 0 | 18 | 158 | 463 | 169 | 0 | 47 | 178 | 467 | 117 | 0 | 15 | 358 | 350 | 84 | 0 | 5 | 180 | 468 | 67 |
| 4 | ……….. | 11.5–13 | 454 | 5 | 5 | 42 | 164 | 201 | 41 | 5 | 65 | 175 | 186 | 22 | 6 | 30 | 281 | 117 | 20 | 0 | 22 | 173 | 186 | 23 |
| 5 | ……….. | 8.5–11 | 275 | 4 | 6 | 48 | 139 | 66 | 15 | 9 | 72 | 133 | 56 | 5 | 7 | 32 | 192 | 37 | 5 | 0 | 24 | 132 | 77 | 7 |
| 6 | ……….. | 6.5–8 | 146 | 3 | 5 | 56 | 61 | 22 | 2 | 10 | 70 | 49 | 17 | 0 | 3 | 39 | 92 | 11 | 1 | 0 | 31 | 67 | 23 | 1 |
| 7 | ……….. | 3.5–6 | 60 | 2 | 11 | 26 | 20 | 2 | 1 | 16 | 26 | 15 | 3 | 0 | 11 | 16 | 32 | 1 | 0 | 4 | 15 | 20 | 2 | 0 |
| 8 | Negative | 0–3 | 10 | 1 | 5 | 5 | 0 | 0 | 0 | 5 | 4 | 1 | 0 | 0 | 4 | 4 | 2 | 0 | 0 | 1 | 5 | 2 | 0 | 0 |
| Total no. (N) | | | 4589 | | 33 | 207 | 654 | 2078 | 1609 | 45 | 308 | 699 | 2222 | 1307 | 31 | 144 | 1433 | 2033 | 936 | 6 | 107 | 709 | 2396 | 978 |
| Corr. (r), Signif. (*p*), Total resp. (n) | | | | | 0.6351 | | 0.0001 | | 4581 | 0.6577 | | 0.0001 | | 4581 | 0.5786 | | 0.0001 | | 4577 | 0.6376 | | 0.0001 | | 4196 |

Columns: 1, Immediate, self-experienced well being; 2, life satisfaction; 3, happiness; 4, fulfillment of needs.

| | | | | | 5 | | | | | 6 | | | | | 7 | | | | | 8 | | | | |
|---|---|---|---|---|---|---|---|---|---|---|---|---|---|---|---|---|---|---|---|---|---|---|---|---|
| | | | **No. of Resp.** | **Score** | **10** | **30** | **50** | **70** | **90** | **10** | **30** | **50** | **70** | **90** | **10** | **30** | **50** | **70** | **90** | **10** | **30** | **50** | **70** | **90** |
| 1 | Positive | 18.5–21 | 1358 | 8 | 0 | 0 | 7 | 512 | 833 | 0 | 0 | 46 | 896 | 382 | 2 | 18 | 239 | 914 | 179 | 0 | 5 | 105 | 529 | 708 |
| 2 | ……….. | 16.5–18 | 1473 | 7 | 0 | 1 | 36 | 840 | 582 | 0 | 1 | 174 | 1090 | 171 | 5 | 43 | 445 | 887 | 86 | 0 | 16 | 192 | 595 | 638 |
| 3 | ……….. | 13.5–16 | 813 | 6 | 0 | 1 | 61 | 535 | 200 | 0 | 4 | 213 | 519 | 41 | 9 | 48 | 340 | 383 | 24 | 1 | 37 | 144 | 360 | 256 |
| 4 | ……….. | 11.5–13 | 454 | 5 | 0 | 3 | 98 | 297 | 46 | 0 | 6 | 198 | 225 | 13 | 4 | 48 | 244 | 155 | 3 | 1 | 28 | 94 | 207 | 109 |
| 5 | ……….. | 8.5–11 | 275 | 4 | 0 | 10 | 99 | 144 | 15 | 1 | 14 | 139 | 103 | 4 | 4 | 52 | 159 | 56 | 0 | 3 | 21 | 84 | 107 | 47 |
| 6 | ……….. | 6.5–8 | 146 | 3 | 3 | 11 | 58 | 62 | 1 | 0 | 21 | 88 | 31 | 0 | 4 | 54 | 70 | 15 | 2 | 1 | 28 | 50 | 44 | 13 |
| 7 | ……….. | 3.5–6 | 60 | 2 | 1 | 17 | 29 | 8 | 0 | 0 | 8 | 41 | 9 | 0 | 1 | 28 | 28 | 2 | 0 | 3 | 10 | 18 | 18 | 4 |
| 8 | Negative | 0–3 | 10 | 1 | 2 | 4 | 4 | 0 | 0 | 0 | 3 | 5 | 0 | 0 | 1 | 5 | 4 | 0 | 0 | 1 | 2 | 2 | 1 | 2 |
| Total no. (N) | | | 4589 | | 6 | 47 | 392 | 2398 | 1677 | 1 | 57 | 904 | 2873 | 611 | 30 | 296 | 1529 | 2412 | 294 | 10 | 147 | 689 | 1861 | 1777 |
| Corr. (r), Signif. (*p*), Total resp. (n) | | | | | 0.5995 | | 0.0001 | | 4520 | 0.5867 | | 0.0001 | | 4446 | 0.5099 | | 0.0001 | | 4561 | 0.3851 | | 0.0001 | | 4484 |

Columns: 5, Experience of objective, temporal domains (family, work, leisure); 6, experience of objective, spatial domains (self, others, world); 7, expression of life's potentials; 8, objective factors.

### Table of Mean Values

*Average of the group in percentage; no. of resp. in the group*

| | | | % | 1 | | 2 | | 3 | | 4 | | 5 | | 6 | | 7 | | 8 | | | Test (*p* value) | Total QOL |
|---|---|---|---|---|---|---|---|---|---|---|---|---|---|---|---|---|---|---|---|---|---|---|
| 1 | Positive | 18.5–21 | 29.6 | 81.9 | 1358 | 80.7 | 1357 | 76.2 | 1357 | 76.4 | 1302 | 81.5 | 1352 | 73.9 | 1324 | 68.4 | 1352 | 78.4 | 1347 | | **0.0001 | 77.7 |
| 2 | ……….. | 16.5–18 | 32.1 | 75.9 | 1472 | 73.3 | 1471 | 68.8 | 1470 | 70.8 | 1359 | 77.3 | 1459 | 68.9 | 1436 | 63.5 | 1466 | 75.4 | 1441 | | **0.0001 | 72.7 |
| 3 | ……….. | 13.5–16 | 17.7 | 69.4 | 808 | 66.2 | 809 | 62.5 | 807 | 65.0 | 720 | 73.0 | 797 | 64.3 | 777 | 58.7 | 804 | 70.5 | 798 | | **0.0001 | 67.3 |
| 4 | ……….. | 11.5–13 | 9.9 | 60.2 | 453 | 56.8 | 453 | 55.1 | 454 | 58.8 | 404 | 67.8 | 444 | 60.0 | 442 | 54.7 | 454 | 68.2 | 439 | | **0.0001 | 62.0 |
| 5 | ……….. | 8.5–11 | 6.0 | 52.6 | 274 | 48.3 | 275 | 50.1 | 273 | 54.0 | 240 | 62.0 | 268 | 56.1 | 21 | 49.8 | 271 | 63.5 | 262 | | **0.0001 | 56.4 |
| 6 | ……….. | 6.5–8 | 3.2 | 44.5 | 146 | 40.0 | 146 | 45.6 | 146 | 47.2 | 122 | 57.5 | 135 | 50.7 | 140 | 43.7 | 145 | 55.7 | 136 | | **0.0001 | 49.6 |
| 7 | ……….. | 3.5–6 | 1.3 | 35.3 | 60 | 31.7 | 60 | 37.7 | 60 | 37.7 | 41 | 47.1 | 55 | 50.3 | 58 | 39.3 | 59 | 53.7 | 53 | | **0.0001 | 44.0 |
| 8 | Negative | 0–3 | 0.2 | 20.0 | 10 | 22.0 | 10 | 26.0 | 10 | 28.1 | 8 | 33.3 | 10 | 42.3 | 8 | 35.6 | 10 | 52.7 | 8 | | **0.0001 | 36.7 |
| Total no. (N) | | | 100 | 71.9 | 4581 | 69.4 | 4581 | 66.2 | 4577 | 68.3 | 4196 | 74.9 | 4520 | 67.1 | 4446 | 61.4 | 4561 | 73.1 | 4484 | | | 70.1 |

*Deviation from population average as a percentage thereof*

| | | | % | 1 | 2 | 3 | 4 | 5 | 6 | 7 | 8 | | Test (*p* value) | Total QOL |
|---|---|---|---|---|---|---|---|---|---|---|---|---|---|---|
| 1 | Positive | 18.5–21 | 29.6 | 13.9 | 16.3 | 15.2 | 11.8 | 8.8 | 10.2 | 11.4 | 7.3 | | **0.0001 | 11.0 |
| 2 | ……….. | 16.5–18 | 32.1 | 5.5 | 5.7 | 4.0 | 3.7 | 3.2 | 2.7 | 3.4 | 3.1 | | **0.0001 | 3.8 |
| 3 | ……….. | 13.5–16 | 17.7 | –3.5 | –4.6 | –5.6 | –4.9 | –2.5 | –4.2 | –4.4 | –3.5 | | **0.0001 | –4.0 |
| 4 | ……….. | 11.5–13 | 9.9 | –16.3 | –18.0 | –16.8 | –14.0 | –9.5 | –10.6 | –10.9 | –6.8 | | **0.0001 | –11.7 |
| 5 | ……….. | 8.5–11 | 6.0 | –26.8 | –30.4 | –24.3 | –21.0 | –17.3 | –16.5 | –19.0 | –13.1 | | **0.0001 | –19.6 |
| 6 | ……….. | 6.5–8 | 3.2 | –38.1 | –42.3 | –31.0 | –31.0 | –23.3 | –24.4 | –28.9 | –23.8 | | **0.0001 | –29.3 |
| 7 | ……….. | 3.5–6 | 1.3 | –50.9 | –54.3 | –43.1 | –44.9 | –37.1 | –25.1 | –36.0 | –26.6 | | **0.0001 | –37.3 |
| 8 | Negative | 0–3 | 0.2 | –72.2 | –68.3 | –60.7 | –58.8 | –55.5 | –37.0 | –42.0 | –27.9 | | **0.0001 | –47.8 |

Test: If H0: m1 = m2 = ... = mn is rejected, the groups are tested individually: H0: mi = mnon-i.

## Covariation in QOL

*Numbers in percent*

| | | 1 | 2 | 3 | Subjective (1–3 | 4 | 5 | 6 | 7 | Existential (4–7) | Objective 8 | Total QOL (1–3,4–7,8) |
|---|---|---|---|---|---|---|---|---|---|---|---|---|
| Covariation | Δ1 | 86.1 | 84.6 | 75.9 | 82.2 | 70.6 | 64.3 | 47.2 | 53.4 | 58.9 | 35.2 | 58.8 |
| Covariation | Δ2 | 83.2 | 84.8 | 70.9 | 79.6 | 68.5 | 60.1 | 44.7 | 54.4 | 57.3 | 38.5 | 60.0 |
| | ±ε | ±7.0 | ±7.4 | ±7.7 | ±6.1 | ±6.3 | ±4.9 | ±5.6 | ±7.2 | ±4.4 | ±8.0 | ±4.4 |

Δ1 is the measured max-min difference. Δ2 is the variation calculated by weight-modified linear regression; ±ε being the measurement error at α = 0.05.
